# Chronic Stress and Risks for Myocardial Infarction in U.S. Adults

**DOI:** 10.1093/geroni/igaa057.1270

**Published:** 2020-12-16

**Authors:** Heather Farmer, Hanzhang Xu, Ann Marie Navar, Michael Nanna, Linda George, Matthew Dupre

**Affiliations:** 1 Duke University, Durham, North Carolina, United States; 2 Duke University School of Medicine, Durham, North Carolina, United States

## Abstract

Long-term exposure to stress has been linked to multiple behavioral and biological responses that are detrimental to cardiovascular health, but the association between chronic stress and risks for acute myocardial infarction (MI) remains unknown. We examined the association between exposure to chronic stress and MI incidence from 2006 to 2016 using data from a nationally-representative prospective cohort study of adults aged 45 and older (n=15,109). Chronic stressors included ongoing issues related to personal health, social relationships, financial strain, housing, and caregiving responsibilities. Cox proportional hazards models were used to examine the association between the number of chronic stressors and MI while adjusting for confounding risk factors. More than half of the respondents reported ≥2 chronic stressors at baseline. Risks for MI increased incrementally from 1 chronic stressor (HR=1.28; 95% CI, 1.20-1.37) to ≥4 chronic stressors (HR = 2.71; 95% CI, 2.08-3.53) compared with those who reported no stressors. These risks were partly attenuated after adjustments for socioeconomic, psychosocial, behavioral, and clinical risk factors. The impact of chronic stressors was especially pronounced among adults with a history of MI (P value for interaction=.032). In adults with a prior MI, risks for a recurrent MI increased substantially from 1 chronic stressor (HR=1.31; 95% CI, 1.10-1.55) to ≥4 chronic stressors (HR = 2.92; 95% CI, 1.47-5.82) compared to those with no stressors. Chronic stress is a significant risk factor for acute coronary events in U.S. adults. More research is required to further understand the psychosocial, behavioral, and biological mechanisms underlying this association.

